# Framework for the Design Engineering and Clinical Implementation and Evaluation of mHealth Apps for Sleep Disturbance: Systematic Review

**DOI:** 10.2196/24607

**Published:** 2021-02-17

**Authors:** Melissa Aji, Christopher Gordon, Elizabeth Stratton, Rafael A Calvo, Delwyn Bartlett, Ronald Grunstein, Nick Glozier

**Affiliations:** 1 Central Clinical School Faculty of Medicine and Health The University of Sydney Sydney Australia; 2 CIRUS, Centre for Sleep and Chronobiology Woolcock Institute of Medical Research Glebe Australia; 3 Susan Wakil School of Nursing and Midwifery Faculty of Medicine and Health The University of Sydney Sydney Australia; 4 Dyson School of Design Engineering Imperial College London London United Kingdom; 5 Charles Perkins Centre - RPA Clinic Royal Prince Alfred Hospital Sydney Australia; 6 Brain and Mind Center The University of Sydney Camperdown Australia

**Keywords:** mobile applications, sleep, insomnia, internet-based intervention, mHealth, mobile health, systematic review

## Abstract

**Background:**

Mobile health (mHealth) apps offer a scalable option for treating sleep disturbances at a population level. However, there is a lack of clarity about the development and evaluation of evidence-based mHealth apps.

**Objective:**

The aim of this systematic review was to provide evidence for the design engineering and clinical implementation and evaluation of mHealth apps for sleep disturbance.

**Methods:**

A systematic search of studies published from the inception of databases through February 2020 was conducted using 5 databases (MEDLINE, Embase, Cochrane Library, PsycINFO, and CINAHL).

**Results:**

A total of 6015 papers were identified using the search strategy. After screening, 15 papers were identified that examined the design engineering and clinical implementation and evaluation of 8 different mHealth apps for sleep disturbance. Most of these apps delivered cognitive behavioral therapy for insomnia (CBT-I, n=4) or modified CBT-I (n=2). Half of the apps (n=4) identified adopting user-centered design or multidisciplinary teams in their design approach. Only 3 papers described user and data privacy. End-user acceptability and engagement were the most frequently assessed implementation metrics. Only 1 app had available evidence assessing all 4 implementation metrics (ie, acceptability, engagement, usability, and adherence). Most apps were prototype versions (n=5), with few matured apps. A total of 6 apps had supporting papers that provided a quantitative evaluation of clinical outcomes, but only 1 app had a supporting, adequately powered randomized controlled trial.

**Conclusions:**

This is the first systematic review to synthesize and examine evidence for the design engineering and clinical implementation and evaluation of mHealth apps for sleep disturbance. The minimal number of apps with published evidence for design engineering and clinical implementation and evaluation contrasts starkly with the number of commercial sleep apps available. Moreover, there appears to be no standardization and consistency in the use of best practice design approaches and implementation assessments, along with very few rigorous efficacy evaluations. To facilitate the development of successful and evidence-based apps for sleep disturbance, we developed a high-level framework to guide researchers and app developers in the end-to-end process of app development and evaluation.

## Introduction

Sleep disturbance is extremely prevalent and affects 33%-45% of adults [1]. Insomnia is the most common sleep disorder, defined as a chronic and persistent difficulty falling asleep, maintaining sleep, or waking up too early [2]. When left untreated, insomnia significantly increases the risk of adverse health outcomes, including mental health disorders [3,4], cardiovascular disease [5], hypertension [6], and diabetes [7]. As insomnia poses serious risks to mental and physical health, exploring the efficacy of treatment is imperative. Cognitive behavioral therapy for insomnia (CBT-I) is an effective gold standard treatment that has consistently shown moderate to large treatment effects [8-10]. A limitation to the widespread use of CBT-I has been a shortage of adequately trained practitioners to treat the high volume of patients with insomnia.

One potential avenue for addressing these challenges is the use of digital therapy. The widespread adoption of mobile phones and apps can change the delivery of health care. This emerging field, known as mobile health (mHealth), refers to the provision of health care services and practice delivered using mobile technology. mHealth apps provide unique benefits in delivering health information and interventions, given the ubiquity, convenience, and affordability of mobile phones. Over 325,000 mHealth apps are available on app stores, and this number continues to grow rapidly [11]. Research supports the utility of mHealth apps for a range of health issues, including depression, anxiety, schizophrenia, cardiac disease, physical activity, and diabetes [12-16]. The potential of mHealth is particularly significant for sleep disturbances as it presents a promising method for addressing this public health burden.

Health outcomes are not only dependent on the intervention delivered but also on the design engineering process employed. Design engineering combines design thinking, participatory design practices, software engineering methods, software, and quality assurance methods. Without analyzing this process, it is not possible to make inferences about the reasons for failure (eg, low engagement, lack of clinical efficacy) of an app. Alongside, this process involves the incorporation of clinically relevant content that either provides adjunctive or standalone therapy to traditional medical and psychological practice.

Clinical implementation requires consideration of end-user privacy and security, a major concern in mHealth. Recent work has demonstrated that mHealth apps routinely share and commercialize end-user data with third parties with very little transparency [17]. In addition, there are several important regulatory considerations required to protect the public and ensure that mHealth apps meet the minimum requirements of quality. Regulatory bodies for mHealth include HIPAA (Health Insurance Portability and Accountability Act), which is a federal law mandating privacy and security standards, and the FDA (Food and Drug Administration), which evaluates the safety and marketing claims of mHealth apps. In the absence of oversight from these regulatory bodies, it is possible to misuse health care–related data [18].

Despite the potential and ubiquity of mHealth apps, most apps lack evidence for their clinical efficacy among end users [15]. Compounding this problem is a lack of framework to inform and standardize the process and reporting of design, development, and evaluation of mHealth apps [19]. This may lead to clinical inefficacy, lack of medical condition–specific content, poor patient engagement, or even harmful apps [20,21].

Although various research groups have established frameworks for apps for posttraumatic stress disorder (PTSD) [22], bipolar disorder [23], and hypertension [24], there may be differences in the processes used according to the health condition, and there are no existing frameworks for sleep apps. This systematic review aims to assess the extent and nature of the peer-reviewed evidence and proposes a high-level framework for the design engineering and clinical implementation and evaluation of mHealth apps for sleep disturbance.

## Methods

### Search Strategy

This study uses PRISMA (Preferred Reporting Items for Systematic Reviews and Meta-analyses) guidelines [25]. A systematic literature search was conducted using the electronic databases MEDLINE, Embase, Cochrane Library, PsycINFO, and CINAHL for relevant papers published from the inception of the databases through February 2020. Keywords related to sleep and mHealth were searched. To increase the coverage of relevant databases, we conducted a manual search of the Journal of Medical Internet Research and the Journal of Internet Interventions as well as the reference list of retrieved publications. See Multimedia Appendix 1 for an example of the search strategy.

### Inclusion Criteria

The inclusion criteria consisted of peer-reviewed publications that (1) focused on mHealth apps that aimed to measure, track, or improve sleep; (2) described the design engineering, clinical implementation, or clinical evaluation of the apps; (3) focused on an app solely targeting sleep; and (4) targeted adults aged between 18 years and 60 years. The included studies were published in English.

### Exclusion Criteria

Studies were excluded if they (1) focused on a sleep disorder other than insomnia; (2) focused on an app that provided multimodal interventions targeting health aspects other than sleep; (3) described internet, telephone, or text messaging interventions; or (4) were review papers. We excluded review papers that were at the early stages of screening; however, given the difficulty of discerning these papers, most were excluded in the full-text screening.

### Screening

After duplicates were removed, a single author (MA) screened all the titles to identify potentially relevant studies. Abstract and title screen and full-text of potentially relevant studies were reviewed independently by 2 authors (MA and ES). Where there were conflicts, discrepancies were discussed, and consensus was reached with the senior author (NG).

### Data Extraction and Coding

A data extraction template was constructed by one of the authors (MA) to summarize the following characteristics of the studies: first author name, publication date, country, objective, study design, and sample. Full papers were then imported into NVivo (QSR International Pty Ltd, version 11.4.3, 2017) for detailed data extraction and thematic coding. A coding framework was developed by the authors for each of the following categories: design engineering and clinical implementation and evaluation. Relevant segments of text were coded and extracted into a Microsoft Excel spreadsheet. Risk of bias was not assessed, as this was deemed irrelevant to the aims of the paper.

Data were extracted and coded into the following subgroups:

- Design engineering: This stage considers the therapeutic approach, design method, and features and functionalities of the apps. Although we appreciate the design engineering process as an ongoing and iterative process, for the purpose of this paper, we propose that this stage covers the point up until the prototype app is implemented among end users.
- Clinical implementation: This stage refers to the testing of the app among end users and includes an assessment of app maturity, implementation metrics (acceptability, engagement, usability, and adherence), and privacy (ie, data acquisition, use or disclosures of identifiable health data) and regulatory (eg, HIPAA) requirements. To categorize the implementation metrics used by the papers and uniformly compare evidence in the included studies, we developed definitions based on previous literature and adjusted for use with sleep apps [26-29]. Acceptability refers to how intended recipients react to the intervention, for example, interest, user satisfaction, and perceived appropriateness. Engagement refers to the usage metrics of the app. Usability refers to whether the app can be used as intended. Adherence refers to the degree to which the user follows the program and the prescribed recommendations of the therapy.
- Clinical evaluation: This stage refers to the assessment of treatment outcomes among end users.

## Results

### Search Results

**Figure 1:**
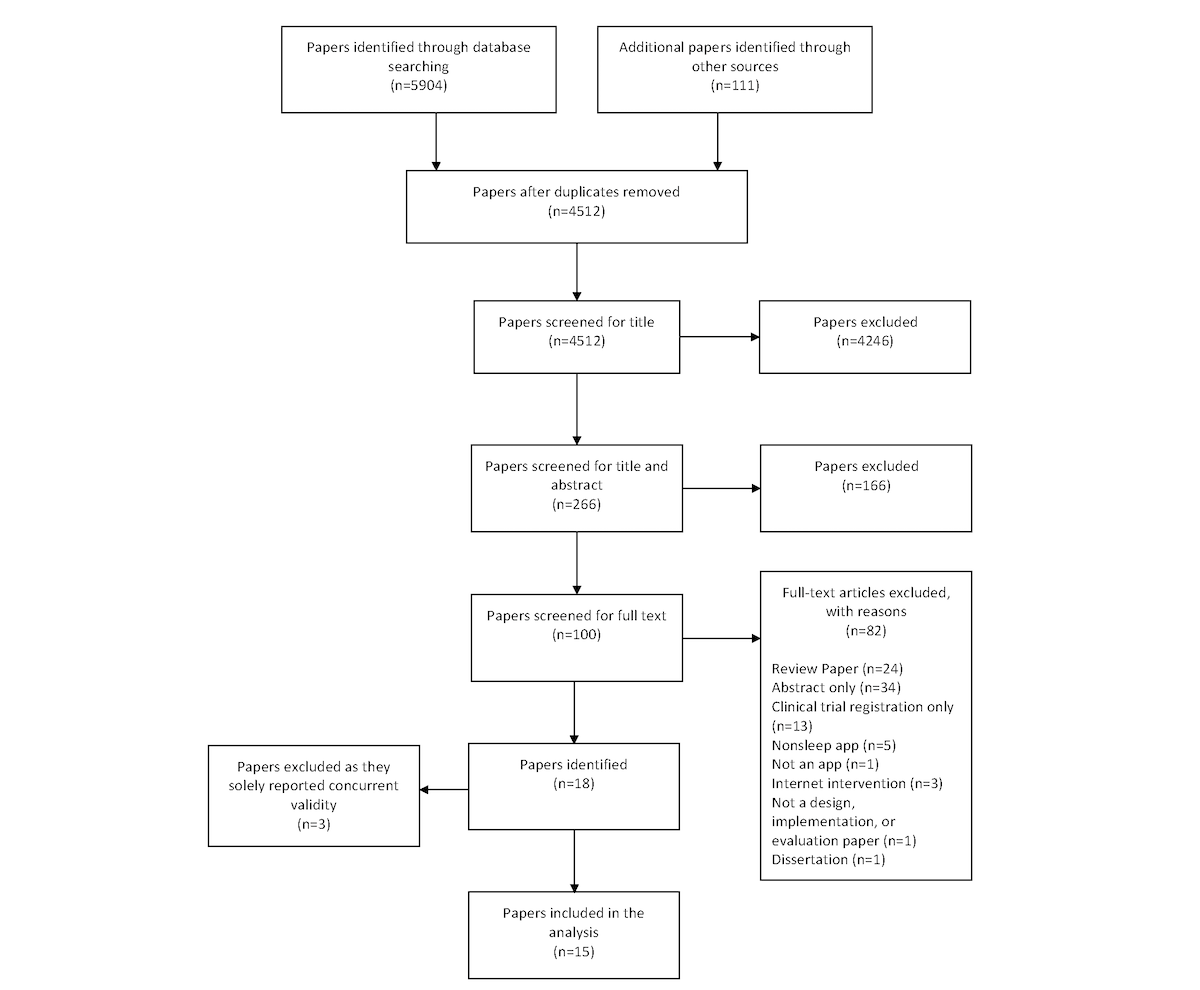
PRISMA (Preferred Reporting Items for Systematic Reviews and Meta-analyses) flow diagram.

The search strategy identified 6015 papers (Figure 1). Following the removal of duplicates (n=1503), 4512 papers were screened for title. Of those, 4246 were excluded, leaving 266 potentially relevant papers. Following a duplicate independent abstract review, 166 papers were excluded. The full text of 100 papers was obtained and independently reviewed by 2 authors (MA and ES). Following this, 82 papers were excluded, primarily for being review papers (n=24) or abstracts only (n=34), leaving 18 full-text papers to be included in this systematic review. A further 3 papers were excluded that reported evidence solely for concurrent validity *(ie, comparison of app sleep tracking against objective measurements of sleep)* and provided no further information about design engineering and clinical implementation and evaluation.

### Study Characteristics

A summary of the study characteristics is presented in Table 1. There was a total of 15 papers. These papers assessed the design, implementation, and evaluation of 8 individual apps.

Furthermore, there were multiple studies (n=27) included within these 15 papers: 7 pre-post studies, 5 cross-sectional studies, 5 mixed methods studies, 5 concurrent validity studies, 2 randomized controlled trials (RCTs), and 3 descriptive studies (Table 1). Table 1 presents the papers and corresponding studies numbered under the column *Number of studies and their objectives.* Subjects were veterans or military service members (n=5) [30-34], healthy participants (n=6) [35-40], individuals with insomnia or sleep disturbances (n=4) [41-44], veterans affairs CBT-I clinicians (n=2) [45,46], and nurses with insomnia (n=1) [47]. The sample size varied among studies, ranging from 2 to 176. For longitudinal studies with over 10 participants, the mean study attrition rate was 25% (range 16%-39%).

**Table 1:** Overview of the included studies.

Study	Country of origin	App name	Sample	Number of studies and their objectives	Study design
Aji et al (2019) [42]	Australia	SleepFix	Participants with insomnia or sleep disturbance	#1-3 - Assess the needs and preferences of those with poor sleep and insomnia	#1- Cross-sectional: survey #2 - Cross-sectional: focus-groups #3 - Cross-sectional: analysis of app reviews
Aji et al (2020) [43]	Australia	SleepFix	Participants with insomnia	#1 - Examine the engagement, acceptability, and preliminary efficacy among end users of SleepFix	#1 - Mixed methods: pre-post and interview
Babson et al (2015) [34]	United States	CBT-I^a^ Coach	Veterans with sleep disturbance and cannabis use disorder	#1 - Examine the acceptability, usability, engagement, and preliminary efficacy among end users of CBT-I Coach	#1 - Pre-post
Baron et al (2018) [39]	United States	Sleep Bunny	Healthy participants with sleep duration <7 hours	#1 - Examine the acceptability and adherence with the prototype version among end users of Sleep Bunny #2 - Examine the acceptability and adherence with the final version among end users of Sleep Bunny	#1 - Mixed methods: pre-post and interview #2 - Mixed methods: pre-post and interview
Bauer et al (2012) [40]	United States	ShutEye	Health participants	#1 - Describe ShutEye app #2 - Examine the acceptability and adherence among end users of ShutEye	#1 - Descriptive report #2 - Mixed methods: pre-post and interview
Horsch et al (2017) [44]	The Netherlands	Sleepcare	Participants with mild insomnia	#1 - Assess the efficacy of Sleepcare	#1 - RCT^b^
Kang et al (2017) [41]	Korea	Unnamed	Participants with insomnia	#1 - Evaluate the efficacy of simplified group CBT-I delivered using a mobile app	#1 - Pre-post
Koffel et al (2016) [33]	United States	CBT-I Coach	Referrals from veterans affair medical center	#1 - Examine the acceptability, adherence, engagement, and preliminary efficacy among end users of CBT-I Coach	#1 - RCT
Kuhn et al (2016) [45]	United States	CBT-I Coach	Veterans affairs CBT-I clinicians	#1 - Describe CBT-I Coach app #2 - Assess acceptability of the app with clinicians (before and after usage)	#1 - Descriptive report #2 - Cross-sectional surveys
Miller et al (2019) [46]	United States	CBT-I Coach	Veterans affairs CBT-I clinicians	#1 - Assess acceptability of the app with clinicians (after app usage)	#1 - Cross-sectional survey
Omeogu et al (2020) [47]	United States	CBT-I Coach	Female veterans administration nurses with insomnia	#1 - Examine the efficacy of CBT-I Coach	#1 - Pre-post
Pulantara et al (2018) [31]	United States	iREST^c^	Active duty service members and veterans	#1 - Describe iREST #2 - Examine usability among end users of iREST #3 - Assess the concurrent validity of iREST against a wearable	#1 - Descriptive report #2- Pre-post #3 - Comparison of app sleep diary against Fitbit
Pulantara et al (2018) [30]	United States	iREST	Military service members and veterans	#1 - Evaluate the efficacy of just-in-time adaptive intervention	#1 - Pre-post
Reilly et al (2019) [32]	United States	CBT-I Coach	Veterans with insomnia	#1 - Evaluate the efficacy of CBT-I Coach	#1 - Pre-post
Shirazi et al (2013) [35]	Germany	Somnometer	University students	#1 - Assess the acceptability, engagement, and usability of the Somnometer app #2 - Assess the concurrent validity of Somnometer #3 - Collect user’s app usage patterns in-the-wild	#1 - Mixed methods: pre-post and interview #2 - Comparison of app against actigraphy #3 - Pre-post

^a^CBT-I: cognitive behavioral therapy for insomnia.

^b^RCT: randomized controlled trial.

^c^iREST: Interactive Resilience Enhancing Sleep Tactics.

### Design Engineering

Table 2 summarizes the 8 individual apps identified in this review: CBT-I Coach [33,34,45,46], Somnometer [35], Interactive Resilience Enhancing Sleep Tactics (iREST) [30,31], ShutEye [40], Sleepcare [44], Sleep Bunny [39], SleepFix [42,43], and 1 unnamed app [41].

**Table 2:** Design characteristics of the included mobile apps.

App name	Therapeutic approach	Design method	Level of automation
CBT-I^a^ Coach	CBT-I including sleep restriction therapy, stimulus control, psychoeducation, cognitive restructuring, and relapse prevention	•Multidisciplinary team, including experts in insomnia, VA^b^-trained CBT-I clinicians, clinical intervention development, technology, and implementation science • Clinician involvement in design	Fully automated. However, it is not designed to replace clinician-delivered CBT-I; nonetheless, the app can be used as an educational resource
iREST^c^	Traditional behavioral insomnia workflows, brief behavioral therapy for insomnia, a military study, and previous implementation of the JITAI^d^ platform	• Clinician involvement in design • Developed based on design principles: iterative and incremental development software model	High clinician input regarding treatment, information, recommendations, and messaging
ShutEye	Mobile, peripheral display to provide real-time sleep hygiene recommendations, relevant to the users’ set bed and wake time goals	N/A^e^	Fully automated
Sleepcare	CBT-I including sleep restriction, relaxation, and sleep hygiene	• End-user involvement • Developed based on persuasive strategies: talk-and-tool design principle	Fully automated
SleepFix	Sleep restriction therapy and stimulus control	• End-user involvement • Multidisciplinary team	Fully automated
Sleep Bunny	CBT-I and motivational interviewing and a telephone coaching manual developed from a web-based depression intervention	N/A	Fully automated
Somnometer	Social alarm clock app. Users rate their sleep quality and specify their sleep status, which they can share with their social network. Sleep duration is estimated based on the user’s phone interaction with app	N/A	Fully automated
Unnamed	CBT-I including sleep restriction therapy and stimulus control therapy	N/A	Clinician input required

^a^CBT-I: cognitive behavioral therapy for insomnia.

^b^VA: veterans affairs.

^c^iREST: Interactive Resilience Enhancing Sleep Tactics.

^d^JITAI: just-in-time adaptive intervention.

^e^N/A: not applicable.

#### Therapeutic Approach

A total of 4 apps were identified to deliver a CBT-I intervention, 1 app used behavioral therapy for insomnia (BTi), 1 app delivered sleep restriction therapy (SRT), 1 app was a social alarm clock, and 1 app was a wallpaper display to promote healthy sleep behaviors. The average length of intervention (ie, use of app in the paper) was 5 weeks (mean 4.5. SD 1.5; median 4.5, range 3-6.5 weeks).

#### Design Approach

The apps were developed using various design approaches, including multidisciplinary (n=2), clinician involvement (n=2), user-centered (n=1), talk-and-tool design principle (n=1), and iterative and incremental development software model (n=1; Table 2). A total of 4 apps did not specify their design approach in their corresponding papers.

#### Features and Functionality

Table 3 presents the features and functionality of the apps. All apps provided personalized sleep feedback (8/8, 100%). Most apps included psychoeducation or sleep hygiene (7/8, 88%), a sleep diary (6/8, 75%), reminders (5/8, 63%), and visualization features (5/8, 63%). In total, 5 of the 8 apps were fully automated and 3 required clinician input. Papers describing 6 apps provided end-user feedback regarding design [31,33,35,39,40,43]. End-user feedback was generally positive in nature, although some papers reported some negative feedback in the design process (4/6, 67%) [31,39,40,43].

**Table 3:** App features and functionalities included in the apps (N=8).

Feature	Apps in which the features were included, n (%)	Apps
Personalized sleep feedback	8 (100)	CBT-I^a^ Coach, iREST^b^, ShutEye Sleepcare, SleepFix, Sleep Bunny, Somnometer, and unnamed app
Psychoeducation or sleep hygiene	7 (88)	CBT-I Coach, iREST, ShutEye, Sleepcare, SleepFix, Sleep Bunny, and unnamed app
Sleep diary	6 (75)	CBT-I Coach, iREST, Sleepcare, SleepFix, Sleep Bunny, and unnamed app
Reminders	5 (63)	CBT-I Coach, iREST, Sleepcare, Sleep Bunny, and SleepFix
Visualization	5 (63)	CBT-I Coach, iREST, SleepFix, Sleep Bunny, and Somnometer
Wearable synchronization	4 (50)	iREST, SleepFix, Sleep Bunny, and unnamed app
Relaxation	3 (38)	CBT-I Coach, Sleepcare, and unnamed app
Alarm	2 (25)	CBT-I Coach, and Somnometer
Clinician portal	2 (25)	iREST and unnamed app
Social features	2 (25)	SleepFix and Somnometer
Messaging with clinician	1 (13)	iREST

^a^CBT-I: cognitive behavioral therapy for insomnia.

^b^iREST: Interactive Resilience Enhancing Sleep Tactics.

### Clinical Implementation

#### Implementation Metrics

All 8 apps included in this review had at least one paper assessing an implementation metric. A total of 10 out of the 15 papers reported on one or more of 4 implementation metrics: acceptability, usability, adherence, and engagement (Table 4).

**Table 4:** Attributes related to implementation in included papers (N=15).

Attribute	Papers assessing attribute, n (%)	Described apps	Measures
Acceptability by end user	6 (40)	CBT-I^a^ Coach, ShutEye, Sleep Bunny, SleepFix, and unnamed app	Interview [33,35,40,43], survey [35,39,41], and MAUM^b^ questionnaire [34]
Acceptability by clinician	2 (13)	CBT-I Coach	17-item measure of clinicians’ retrospective perceptions of app [45] and 35-item measure of clinicians’ retrospective perceptions of app [46]
Engagement	6 (40)	CBT-I Coach, iREST^c^, SleepFix, Somnometer, and unnamed app	Self-reported number of sleep diaries [33,41]; MAUM questionnaire measuring frequency of app use and length of use per session [34]; number of sleep diary entries, mood logger entries, and game sessions measured by the app [43]; number of scheduled alarms, posts shared on Facebook, and sessions measured by the app [35]; and number of days the app was used, completion time for each log, number of devices with app accessing server each day measured by the app [31]
Usability	4 (27)	CBT-I Coach, iREST, ShutEye, SleepFix, and Somnometer	System Usability Scale [31,35,43], modified Telerehabilitation Usability Questionnaire [31], MAUM questionnaire [34], qualitative survey [31], and qualitative interview [40]
Adherence	3 (20)	CBT-I Coach, ShutEye, Sleep Bunny, and Sleepcare	Average number of days and time spent on homework [33], patient adherence form (completed by clinicians) [33], number of relaxation exercises performed [44], deviation between real and agreed-upon time in bed [44], number of coaching sessions completed [39], and qualitative interview [40]

^a^CBT-I: cognitive behavioral therapy for insomnia.

^b^MAUM: Mobile App Use Measure.

^c^iREST: Interactive Resilience Enhancing Sleep Tactics.

Acceptability among end users and engagement were the most frequently assessed implementation metrics. Most papers measured acceptability through an interview or survey with end users and engagement using app-measured or self-reported app usage metrics. Usability was measured by less than half of the papers, with most using the System Usability Scale. Most apps had evidence available for 1 to 2 implementation metrics, and only 3 out of 8 apps (CBT-I Coach, ShutEye, and SleepFix) had papers reporting more than 2 implementation metrics.

#### App Maturity

The maturity levels of the apps ranged on a continuum of 3 levels: prototype, matured, and released [48]. A prototype refers to a minimally viable product of the app with functionality that users can test. A matured version refers to an app that has undergone user testing and has been redesigned. A released version refers to an app that is available for download. Most apps were prototypes (n=5), and 1 app was a prototype-to-mature version (Table 5). Only 1 app was matured to release (CBT-I Coach). Furthermore, 1 study had no information regarding the maturity level of the app described. Only CBT-I Coach was available from the Google Play or Apple App Store at the time of writing the primary paper.

**Table 5:** Maturity of the included apps.

Stage of maturity	App
Prototype	iREST^a^, ShutEye, Sleep Bunny, SleepFix, and Somnometer
Prototype to matured	Sleepcare
Matured	—^b^
Matured to released	CBT-I^c^ Coach
Released	—
No information	Unnamed [41]

^a^iREST: Interactive Resilience Enhancing Sleep Tactics.

^b^No apps met this maturity level.

^c^CBT-I: cognitive behavioral therapy for insomnia.

Only 3 apps considered the privacy of data collected [35,40,45]. In total, 3 out of the 4 apps that enabled wearable synchronization reported data relating to its use adjunct to the app [31,41,43]. No studies have considered the regulatory requirements of the apps.

### Clinical Evaluation

In total, 6 of the 8 apps had a quantitative evaluation of treatment outcomes. Of the 11 studies that evaluated clinical effectiveness, the most frequently used sleep outcomes were self-reported sleep questionnaires, followed by app sleep diary measures and actigraphy (objective). In total, 8 of the 11 papers used the Insomnia Severity Index, the most widely used insomnia treatment outcome questionnaire to evaluate insomnia symptom severity [49] (Table 6). Only 2 papers focused solely on evaluating the effectiveness of the app [44,47].

**Table 6:** Clinical outcome measures used in included papers.

Measures			Study
**Sleep**			
	**Self-report questionnaires**		
		Insomnia Severity Index	[30-33,41,43,44,47]
		Pittsburgh Sleep Quality Index	[30,32,34,41,43,44]
		Epworth Sleepiness Scale	[30,39,40,43]
		Dysfunctional Beliefs and Attitudes about Sleep-16	[41,44]
		Functional Outcomes of Sleep Questionnaire	[32]
	**App sleep diary measures**		
		Time in bed	[44]
		Total sleep time	[44]
		Sleep efficiency (percentage of time spent asleep while in bed)	[41,43,44]
		Wake after sleep onset (wake time after initial sleep onset)	[43]
		Sleep onset latency (time taken to fall asleep)	[41,43]
	**Actigraphy (objective)**		
		Sleep efficiency	[39,41]
		Total sleep time	[39,41]
		Stages of sleep	[41]
		Number of awakenings	[32]
	**Fitbit**		
		Wake after sleep onset	[43]
		Sleep efficiency	[43]
**Psychological measures**			
	**Depression and anxiety**		
		Hospital Anxiety and Depression Scale	[43,44]
		Center for Epidemiologic Studies Depression Scale	[44]
		PHQ^a^-8, PHQ-9, or PHQ-15	[30,32]
		Generalized Anxiety Disorder Scale-7	[30]
		Beck Depression Inventory	[41]
		Beck Anxiety Inventory	[41]
	**PTSD^b^**		
		PTSD Checklist Civilian Version	[30,32]
		Pittsburgh Sleep Quality Scale (PSQI-A^c^ for PTSD)	[30]
	**Other**		
		12-item Short Form Survey	[43]
		Flinders Fatigue Scale	[43]
		Clinical Global Impression–Improvement Scale	[30]
		West Haven-Yale Multidimensional Pain Inventory	[32]
		Pain Disability Index	[32]
		Marijuana smoking history	[34]
		Timeline follow-back interview	[34]

^a^PHQ: Patient Health Questionnaire.

^b^PTSD: posttraumatic stress disorder.

^c^PSQI-A: Pittsburgh Sleep Quality Index–Addendum.

## Discussion

### Principal Findings

This study provides the first comprehensive review of published studies and a framework for the design engineering and clinical implementation and evaluation of mobile apps for sleep disturbance. Despite the availability of over 500 [50] sleep mobile apps in commercial app stores (Apple App and Google Play Store), our review identified 15 papers assessing the design, implementation, and evaluation of 8 apps, only one of which was available to download on commercial app stores [32,33,40,45-47]. This means that less than 1% of all commercially available sleep apps have any published data on these aspects. Of the 15 papers, implementation metrics were reported in 10 papers and treatment outcomes were evaluated in 11 papers. Despite the potential of mHealth, there has been a small number of studies, a lack of standardization in design engineering approaches and clinical implementation assessments, and few comprehensive clinical evaluations.

### Design Engineering

For the increased utilization and adoption of mHealth apps, these technologies must be designed for people who will use them—both end users and multidisciplinary stakeholders [51-54]. Our review indicates that although some apps utilized best practice design approaches, for example, user-centered and multidisciplinary methods, approximately half of the apps did not report their design approach. We have previously shown how people with insomnia have unique user needs and preferences for sleep mobile apps that can drive engagement [42]; however, only 2 of the 8 apps reported any end-user involvement. Multidisciplinary teams are particularly crucial in a domain such as sleep, where various stakeholders (eg, clinical psychologists, sleep clinicians, psychiatrists) tend to be involved in patient care; however, only 2 apps reported this approach. Although these best practice design approaches may be an unspoken rule in app development, transparency in the reporting of these approaches is important in encouraging clinicians to be able to recommend such apps designed to sustain engagement.

### Clinical Implementation

There were few comprehensive evaluations of implementation, with only 3 out of 8 apps reporting more than 2 implementation metrics. Poor implementation can limit adoption or engagement, particularly in an uncontrolled and real-world setting, which in turn limits effectiveness. Our results suggest that although most apps had papers reporting some implementation outcomes, very few conducted a comprehensive exploration. For instance, although there is support for Sleepcare’s efficacy, there was a lack of a substantial assessment for its implementation. The National Health Service in the United Kingdom highlights the importance of implementation in the acknowledgment that their previous failure of digital health technology deployment was attributed to rushed and inadequate implementation [55]. Moreover, reporting of implementation processes may enable replication and reduce the gap between research and practice [56].

There was great heterogeneity among implementation studies in the conceptualization of implementation metrics. Given the inconsistency in terminology, we developed our own definitions to facilitate cross-study comparisons. This language incongruence highlights the need for a taxonomy of implementation metrics to clearly delineate key variables [57,58]. Similarly, there was a lack of standardized implementation metrics ranging from qualitative methods to nonstandardized quantitative surveys. This further contributes to blurring among constructs. When standardized measures were used, they were not specifically designed for mobile or sleep disturbances. For instance, the System Usability Scale was one of the most frequently used implementation scales in the identified studies [59]. Although it is a well-researched measure with good psychometrics [59], it is not designed for mobile or sleep use. A taxonomy of implementation outcomes with standardized tools for sleep mobile apps can advance the measurement and understanding of implementation processes.

Despite the importance of privacy and regulatory oversight in digital technology, only 3 papers mentioned privacy, and no papers considered mHealth regulations. Mobile devices collect a large amount of behavioral and health care data, raising concerns regarding mHealth app quality and safety [60-62]. Most mHealth apps do not have a transparent privacy policy, leaving end users unaware of what data are collected, how data are transferred, where data are stored, and with whom the data are shared [63]. Given that data security is a key concern among health care providers when recommending mHealth apps [64], transparent privacy policies and further regulatory oversight from bodies such as the FDA can combat this issue.

### Clinical Evaluation

Of the evaluation studies, we identified 2 RCTs, only one of which was adequately powered according to the primary paper [44]. Most of the apps (6/8, 75%) delivered full or modified CBT-I (SRT or BTi), a well-established intervention where an evidence base already exists for face-to-face and internet-enabled delivery. CBT-I has been recommended as a first-line therapy for insomnia, given its substantial clinical base [8-10], and has shown comparable efficacy when delivered via the internet [65]. Although a previous systematic review demonstrated support for the efficacy of mobile phone interventions for sleep disorders and sleep quality, this review only included studies of 4 mobile apps [66]. In this review, only 2 of these 4 apps were included. One was excluded as it was a multimodal intervention and the other was targeted toward older adults. There is an evident need for methodologically robust and adequately powered studies assessing the effectiveness of mHealth apps for sleep disturbance. Nevertheless, mobile-delivered CBT-I has potential, given the therapy’s existing evidence base across various modalities.

Most apps described in this review were prototype versions, with only one app being matured to released (CBT-I Coach). This is aligned with a greater number of identified studies primarily focusing on earlier stages of development, for example, design or implementation, with some preliminary evaluations of efficacy. Of the apps identified in this review, only 1 app (Sleepcare) has the support of efficacy from an adequately powered RCT [44]. Despite this, there are no implementation studies for Sleepcare, and although acceptance is included as a measure in the paper, the results are not reported. Conversely, several papers described the design and implementation of the CBT-I Coach, but no full-scale efficacy evaluation was identified. However, CBT-I Coach is the only app available in commercial app stores.

Although it might be thought that a more mature app would have more cumulative evidence for its design, implementation, and evaluation, this does not prove to be the case. This mismatch in the maturity of sleep apps and the levels of available evidence ultimately reflects the unregulated nature of mHealth app development and deployment. To exacerbate the problem, the commercial app marketplace allows developers and researchers to freely release apps into these stores, which serve as the main app repository for consumers. Evidently, there is a need for a standardized set of evidence-based criteria for researchers to meet before making commercially available apps.

Ultimately, the lack of standardization in the evidence and reporting for the design engineering and clinical implementation and evaluation of mHealth apps for sleep disturbance stresses the need for a comprehensive framework to guide researchers and app developers. A recent systematic review highlighted the wide heterogeneity among the different published criteria for the assessment of mHealth apps, with 38 main classes of criteria [19]. Guidelines specific to the development and assessment of apps for sleep disturbance are particularly scarce. Establishing an extensive and standardized framework for mobile apps for sleep disturbance may lead to improved existing tools and the development of successful, high-quality, and effective apps.

### mHealth App Framework for Sleep Disturbance

**Figure 2:**
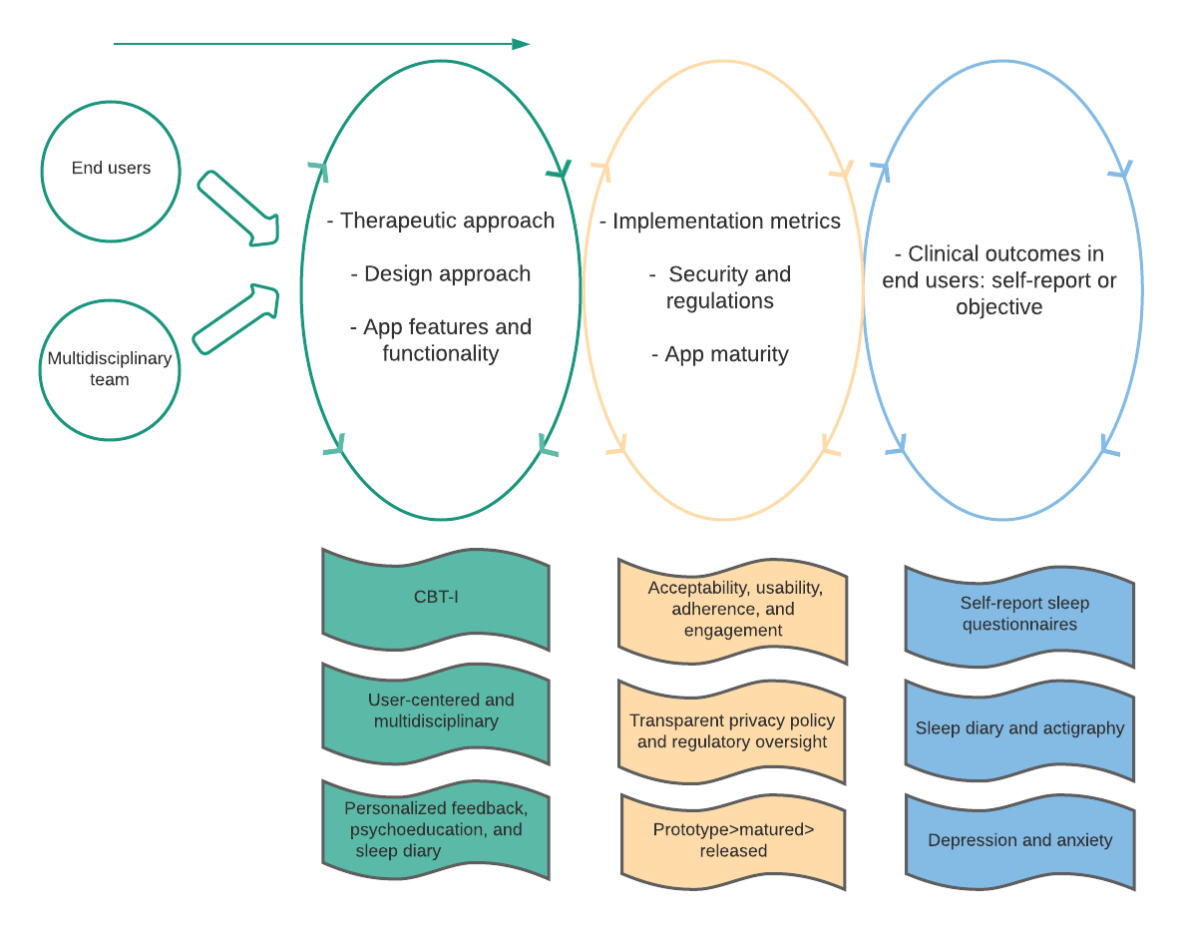
Framework for the design engineering and clinical implementation and evaluation of apps for sleep disturbance. CBT-I: cognitive behavioral therapy for insomnia.

As a first step, we developed a high-level framework based on the findings of this study to guide the design engineering and clinical implementation and evaluation of apps for sleep disturbance. The findings are summarized in Figure 2.

Although several frameworks exist for conditions such as PTSD [22], bipolar disorder [23], and hypertension [24], there are no frameworks for the development of sleep apps. Each of these frameworks similarly address design engineering, clinical implementation, and evaluation. For instance, the framework for bipolar disorder also notes the importance of designing with end users and multidisciplinary teams, addressing security and regulations with standards consistent with HIPAA, and evaluation with end users [23]. These frameworks were developed through a combination of lessons learned from firsthand app development, best practice principles, and theory-based design models. The framework in this study triangulates the findings from this review of digital sleep interventions to these previous frameworks augmented by our firsthand experience with the development of an app for insomnia [43,67]. Future work may consider adapting theory-based design models for sleep disturbance and integrating them into this framework.

Several reviews of commercial sleep apps have demonstrated a lack of validated sleep measurement algorithms [68], evidence-based principles for insomnia management [69], behavior change constructs [70], and overall low quality of functionality and content based on established app assessment criteria [71,72]. Evidently, commercial development of apps has severely outpaced academic research, putting their trustworthiness in question [73]. Our systematic search identified 13 clinical trial registrations, of which 6 were mobile apps not included in our systematic review as there were no available publications. Although partly attributable to the infancy of the mHealth field, there is still a necessity for timely and increased efforts of mobile sleep apps to progress to clinical evaluations. Collaboration between academia and industry may offer an opportunity to work together in developing scientifically rigorous solutions while keeping pace with the rapidly evolving app market.

This review has several limitations. First, we included English language publications only, which introduces publication bias, particularly given that these papers tended to originate from high-income countries such as the United States and Australia. Second, given that data extraction was based on the included studies only and that the mobile apps were not downloaded by the authors, some information such as app features and design approaches was not always clear or available. Third, given that our study focused on apps for sleep disturbance and did not include mHealth apps with multimodal interventions, including sleep, the inferences from this study may not extend to all sleep apps.

### Conclusions

This is the first review to evaluate the design engineering and clinical implementation and evaluation of apps designed for sleep disturbance. It was found that despite a plethora of sleep apps available, there is limited research and a lack of standardization in the evidence base for the design, implementation, and evaluation of apps for sleep disturbance. Few apps had evidence for the use of best practice design approaches. Implementation assessments lacked standardization and consistency in implementation metrics used, and very few comprehensive efficacy evaluations were identified.

For the future development of engaging and evidence-based apps for sleep disturbance, we have developed a framework to guide the development and deployment process. The framework aims to address the need for (1) increased application and reporting of best practice design approaches, for example, user-centered and multidisciplinary teams; (2) comprehensive implementation assessments involving multiple metrics, tools validated for sleep, and privacy and regulatory considerations; and (3) rigorous evaluations of clinical efficacy. Collaboration between academia and the industry may facilitate the development of evidence-based apps in the fast-paced mHealth technology environment.

## Appendix

Multimedia Appendix 1 Example search strategy.

## Abbreviations

BTi: behavioral therapy for insomnia
CBT-I: cognitive behavioral therapy for insomnia
FDA: Food and Drug Administration
HIPAA: Health Insurance Portability and Accountability Act
mHealth: mobile health
PTSD: posttraumatic stress disorder
SRT: sleep restriction therapy
