# Supplementary material for: Framework for the Design Engineering and Clinical Implementation and Evaluation of mHealth Apps for Sleep Disturbance: Systematic Review
Source: J Med Internet Res. 2021 Feb 17;23(2):e24607. doi: 10.2196/24607 (PMC7929739; doi:10.2196/24607)
Supplement: Multimedia Appendix 1 [file jmir_v23i2e24607_app1.docx]

Table 1: Example search strategy terms

**Cochrane**

((sleep*):ab,ti OR (insomnia):ab,ti) AND ((mhealth):ab,ti OR (m-health):ab,ti OR (ehealth):ab,ti OR (e-health):ab,ti OR (mobile health*):ab,ti OR (mobile app*):ab,ti OR (mobile device):ab,ti OR (mobile technolog*):ab,ti OR (mobile phone):ab,ti OR (cell*):ab,ti OR (digital health):ab,ti OR (smart phone):ab,ti OR (smartphone):ab,ti OR ((cell* OR smart OR mobile) adj phone):ab,ti OR (iphone OR ios OR android):ab,ti)"
